
JOURNAL INFORMATION
==============================
NLM Title Abbreviation: Brain Commun
Journal ID: Brain Commun
NLM Journal ID: 101755125
Journal ID: braincomms

Brain Communications

EISSN: 2632-1297

ARTICLE INFORMATION
==============================
PMCID: PMC8195160
PMID: 34169281
DOI: 10.1093/braincomms/fcab118
Article ID: fcab118
Article version: 1
Subjects: Letter to the Editor, AcademicSubjects/MED00310, AcademicSubjects/SCI01870

Gut-brain axis in the neurological comorbidity of COVID-19

Chen Jiezhong 1
Vitetta Luis 1 2
1 Research Department, Medlab Clinical , Sydney, 2015, Australia
2 The University of Sydney , Faculty of Medicine and Health, Sydney, 2006, Australia
Correspondence to: Dr. Jiezhong Chen Email: Jiezhong_chen@medlab.coCorrespondence may also be sent to: Professor Luis Vitetta Email: luis.vitetta@sydney.edu.au
Electronic publication date: 2021 May 27
Electronic Location ID: fcab118
Received 2021 Apr 25; Accepted 2021 May 7
Copyright: © The Author(s) (2021). Published by Oxford University Press on behalf of the Guarantors of Brain.
Copyright year: 2021
License: This is an Open Access article distributed under the terms of the Creative Commons Attribution License (http://creativecommons.org/licenses/by/4.0/), which permits unrestricted reuse, distribution, and reproduction in any medium, provided the original work is properly cited.
License URL: https://creativecommons.org/licenses/by/4.0/

Page count: 5
edited-state: accepted-manuscript
article-lifecycle: PAP

==============================
Letter to the Editor

### Gut-brain axis in the neurological comorbidity of COVID-19

Jiezhong Chen[1], Luis Vitetta[1,2]

1. Research Department, Medlab Clinical, Sydney 2015, Australia
2. The University of Sydney, Faculty of Medicine and Health, Sydney 2006, Australia

Correspondence to:
Dr. Jiezhong Chen Email: Jiezhong_chen@medlab.co

Correspondence may also be sent to:
Professor Luis Vitetta Email: luis.vitetta@sydney.edu.au

© The Author(s) (2021). Published by Oxford University Press on behalf of the Guarantors of Brain.
This is an Open Access article distributed under the terms of the Creative Commons Attribution License (http://creativecommons.org/licenses/by/4.0/), which permits unrestricted reuse, distribution, and reproduction in any medium, provided the original work is properly cited.

Severe COVID-19, characterized by a cytokine storm and lymphopenia, has led to increased levels of mortality due to multiple organ failure.[1] Brain-associated comorbidity with an increased mortality rate develops in approximate one third of COVID-19 patients.[2,3] Thus, further central nervous system considerations and improvement of COVID-19 neurological comorbidity could reduce the morbidity that ensues with COVID-19. A cytokine storm has been recognized to be a major characteristic of COVID-19 neurological comorbidity through CSF analyses.[4,5] In the review article by Meier and colleagues (2021)[6] the emphasis was directed at the importance of the brain cytokine storm in neurodegeneration and cognition, which caused the large loss of labour force productivity. However, how the brain cytokine storm is shaped by COVID-19 infections that is reflected in neurological comorbidity pathophysiological processes, still has to be elucidated. We posit that the involvement of the gut-brain axis in the brain cytokine storm in COVID-19 neurological comorbidity is a significant determinant of COVID-19 severity and is of therapeutic implications.

The intestinal microbiota presents a complex structure that develops throughout life presenting trillions of commensal bacteria that exert multiple beneficial effects on the human host.[7] Dysregulation of the gut microbiota (gut dysbiosis) is associated with metabolic disease in various organs through bidirectional interactions such as happens with the gut-brain axis.[1] Gut dysbiosis alters the inflammatory tone of the gut that leads to chronic inflammation with decreased anti-inflammatory mechanisms. These effects result from reduced abundance and adverse shifts in diversity of the commensal bacterial cohort with subsequent decreases in beneficial metabolites namely, butyrate, bile acid derivatives and tryptophan.[1] The gut-brain axis has been often reported involved in neurological disorders[8,9] and thus the intestinal dysbiosis could be incorporated into the narrative of neurological manifestations of COVID-19. The patients with COVID-19 neurological comorbidity are characterized as those with advanced age, hypertension, renal failure, and neoplastic diseases.[2] All these risk factors are involved with gut dysbiosis. Therefore, gut dysbiosis could be a major contributing factor in the formation of the cytokine storm in the brain. Furthermore, the systemic inflammation from other organs infected by SARS-CoV-2 also contributes to the formation of the cytokine storm in the brain.[1] A study showed that the mortality is highest in those COVID-19 patients with both neurological and other major organ manifestations, followed by those with neurological manifestations only and then by those with no systemic manifestations.[2]

In addition, gut dysbiosis could also involve in the local vascular abnormality to cause fatal thrombosis. The local vascular inflammation is not only caused by SARS-CoV-2 infection but also accelerated by gut dysbiosis-caused systemic inflammation and decreased anti-inflammatory mechanisms.[1] The local vascular infections from SARS-CoV-2 could be important for the formation of hyperinflammation in the brain. Multiple routes have been described for SARS-CoV-2 entry into the brain including trans-synaptic transfer across infected neurons, entry via the olfactory nerve, infection of vascular endothelia and leukocyte migrations across the blood-brain barrier.[10] Importantly, the virus has high affinity for vascular endothelial cells due to the high expression of ACE2 in these cells. The virus-induced damage to endothelial cells together with systemic inflammatory aspects promotes the formation of vascular thrombosis, causing serious adjacent pathophysiological changes. Therefore, even if there is no viral entry into the CSF, the virus could contribute to the brain cytokine storm through vascular thrombosis-caused insults. It is understandable that the most common risk factor for neurological comorbidity is pre-existing cerebrovascular diseases, accounting for 39% of these patients.[2] Gut dysbiosis can lead to increased gut permeability to allow translocation of endotoxins and bacteria to the circulation and extra-intestinal organs. It results in increased systemic inflammation, accelerating vascular thrombosis.[1]

On the other hand, brain pathological changes could further increase gut dysbiosis. It has been well demonstrated that gut-brain axis has bidirectional effects.[9] Gut dysbiosis can cause neurological inflammation while psychological disorders also result in intestinal diseases. Indeed, COVID-19 infections could cause these patients stress, anxiety and depression. These psychological distresses can further increase gut dysbiosis and inflammation. This could accelerate the impact on neurological comorbidity to form feed-forward loop. Studies have shown that psychological distresses could promote mast cells to secret pro-inflammatory cytokines such as IL-6 and TNF-alpha through corticotropin-releasing hormone.[1] Therefore, inflammation is a key link between gut and brain.

An appreciation of the role of intestinal dysbiosis in the cytokine storm in COVID-19 neurological comorbidity has therapeutic implications. Modulation of gut dysbiosis to improve gut microbiota could be achieved by various approaches such as probiotics, prebiotics, fecal microbiome transplantation and dietary modification.[9] Administration of commensal bacterial metabolites such as butyrate could reduce systemic inflammation. The bacterial metabolites such as butyrate and bile acid derivatives can activate regulatory T cells to reduce inflammation caused by SARS-CoV-2 infections.[1] Although corticosteroids can effectively reduce mortality rate, early anti-inflammatory therapy could be effective, avoiding the pronounced side effects with corticosteroid administration. For example, anti-IL6 together with commensal bacterial metabolites could be an effective regimen that warrants further investigation.

**Data availability**

Data sharing is not applicable to this article as no new data were created or analysed.

**Competing interests**

We declare that we have no competing interests.

### References

1. Chen J, Hall S, Vitetta L. Altered gut microbial metabolites could mediate the effects of risk factors in Covid-19. *Rev Med Virol.* 2021; e2211. https://doi.org/10.1002/rmv.2211.

2. Romagnolo A, Balestrino R, Imbalzano G, et al. Neurological comorbidity and severity of COVID-19. *J Neurol.* 2021; 268(3):762-769.

3. Ellul MA, Benjamin L, Singh B, et al. Neurological associations of COVID-19. *Lancet Neurol.* 2020; 19(9):767-783.

4. Pilotto A, Masciocchi S, Volonghi I, et al. SARS-CoV-2 encephalitis is a cytokine release syndrome: evidences from cerebrospinal fluid analyses. *Clin Infect Dis* 2021; doi: 10.1093/cid/ciaa1933.

5. Heming M, Li X, Rauber S, et al. Neurological manifestations of COVID-19 feature T cell exhaustion and dedifferentiated monocytes in cerebrospinal fluid. *Immunity* 2021; 54, 164-175.

6. Meier IB, Teixeira CVL, Tarnanas I, Mirza F, Rajendran L. Neurological and mental health consequences of COVID-19: potential implications for well-being and labour force. *Brain Commun.* 2021; doi:10.1093/braincomms/fcab012.

7. Tremaroli V, Backhed F. Functional interactions between the gut microbiota and host metabolism. *Nature* 2012; 489:242-249.

8. Cryan J, O'Riordan KJ, Sandhu K, Peterson V, Dinan TG. The gut microbiome in neurological disorders. *Lancet Neurol*. 2020; 19:179-94.

9. Powell N, Walker MM, Talley NJ. The mucosal immune system: master regulator of bidirectional gut-brain communications. *Nat Rev Gastroenterol Hepatol.* 2017; 14(3):143-159.

10. Zubair AS, McAlpine LS, Gardin T, Farhadian S, Kuruvilla DE, Spudich S. Neuropathogenesis and neurological manifestations of the coronaviruses in the age of coronavirus disease 2019: a review. *JAMA Neurol.* 2020; 77(8):1018-1027.
